# Histological Dissection of Fusarium-Banana Interaction Using a GFP-Tagged Subtropical Race 4 Strain of *Fusarium oxysporum* f. sp. *cubense* on Banana Cultivars with Differing Levels of Resistance

**DOI:** 10.3390/microorganisms12122472

**Published:** 2024-12-01

**Authors:** Andrew Chen, Ting-Yan Chou, Yi Chen, Sumayyah M. A. Fallatah, Jay Anderson, Jiaman Sun, Harry Cosgrove, Siyuan Gao, Brett J. Ferguson, Amelie Soper, Donald M. Gardiner, Elizabeth A. B. Aitken

**Affiliations:** 1 School of Agriculture and Food Sustainability, The University of Queensland, St. Lucia, QLD 4072, Australia; yanchou423@gmail.com (T.-Y.C.); yi.chen9@uq.net.au (Y.C.); s.fallatah@uq.net.au (S.M.A.F.); jay.anderson@scu.edu.au (J.A.); jiamansun@hotmail.com (J.S.); harry.cosgrove@daf.qld.gov.au (H.C.); amelie.kelly13@yahoo.com.au (A.S.); 2 Integrative Legume Research Group, School of Agriculture and Food Sustainability, The University of Queensland, St. Lucia, QLD 4072, Australia; b.ferguson1@uq.edu.au; 3 Queensland Alliance for Agriculture and Food Innovation, The University of Queensland, St. Lucia, QLD 4072, Australia; donald.gardiner@uq.edu.au

**Keywords:** Fusarium wilt of banana, fluorescent microscopy, tyloses formation, xylem colonisation, pathogen localisation, root and rhizome infection, containment and tolerance

## Abstract

Fusarium wilt, caused by *Fusarium oxysporum* f. sp. *cubense* (*Foc*), poses a significant threat to global banana production. This study used a GFP-tagged subtropical race 4 strain of *Foc* (GFP-*Foc*-STR4) to trace the pathogen’s movement in different banana cultivars. These include a race 4 resistant cultivar FHIA25 and the Cavendish somaclone ‘GCTCV119’, as well as susceptible cultivars including ‘Lady Finger’, ‘FHIA02’, and ‘Williams’ Cavendish. GFP localization revealed that GFP-*Foc*-STR4 was able to infect all tested cultivars, moving from the roots to the rhizome and aerial parts of the plant. Tyloses formation in root and rhizome vasculature, visualised with GFP autofluorescence and confirmed by scanning electron microscopy, was found to restrict *Foc* within the xylem vessels, slowing its spread but not fully preventing infection. This containment mechanism contributes to the host tolerance of ‘FHIA25’ and ‘GCTCV119’, though it does not confer complete immunity. The use of the fluorescently tagged *Foc* strain provides valuable insight into the infection process, and supports efforts in the integrated management of Fusarium wilt of banana.

## 1. Introduction

The banana (*Musa* spp.), an herbaceous plant native to tropical regions of Indomalaya and Australia, is a key global food crop, valued for its flavour and nutritional content. Widely cultivated across the tropics, bananas serve as a staple food in many countries. In 2022, global banana production reached 135 million tons, with exports totalling 24 million tons, valued at approximately $13 billion USD [1].

The Cavendish subgroup, belonging to the triploid banana cultivar group (genome AAA) of *Musa acuminata*, includes the cultivar Williams, which is the most dominant dessert banana in the market, accounting for 47% of global banana production [1]. The over-reliance on a few cultivars and limited genetic diversity makes banana plantations highly vulnerable to pests and diseases, resulting in major outbreaks that constrain production at both commercial and small-scale levels [2]. One such disease is Fusarium wilt of banana (FWB), caused by the soil-borne fungus *Fusarium oxysporum* f. sp. *cubense* (*Foc*). The *Fusarium oxysporum* species complex encompasses numerous plant pathogens affecting economically significant crops. These pathogens are categorised into specialised forms (formae speciales), each defined by their specific plant hosts. Within the specialised form infecting banana (*Foc*), isolates are classified into races based on their pathogenicity to different banana cultivars [3,4,5]. The evolutionary relationships amongst formae speciales of *F. oxysporum* are complex, characterised by a polyphyletic origin and evidence of horizontal gene transfer [5,6].

*Foc* produces three types of asexual spores: short-lived microconidia, macroconidia, and long-lived chlamydospores. All three can infect susceptible banana plants [7]. *Foc* can persist in the soil for decades, surviving as chlamydospores or saprophytes on dead plant tissue [8]. Spores germinate and adhere to banana roots, entering through the epidermis or wounds, then moving into the xylem vessels of roots and rhizomes [9,10]. As *Foc* colonises the plant, it forms extensive mycelial networks that block xylem vessels, impeding nutrient and water transport, causing wilting, and eventually leading to plant death [11]. External symptoms of FWB include pseudostem splitting, leaf yellowing, necrosis, and stunted growth. Although the fungus does not infect banana fruit directly, infected plants yield less fruit [12]. A recent study has shown that *Foc* race 4 can colonise the fruit peduncle of infected banana plants [13]. This highlights that despite the infection, improvements in agronomic practices have allowed affected plants to continue producing bunches of bananas. This ability to produce fruit, even in the presence of *Foc* tropical race 4 (TR4), suggests potential management strategies for maintaining banana production in regions affected by FWB caused by TR4.

*Foc*, particularly *Foc* race 1 and *Foc* race 4, has had a significant impact on global banana production. *Foc* race 1 caused the collapse of the ‘Gros Michel’ banana (AAA)-dominated banana trade in the 1950s, leading to the widespread replacement of ‘Gros Michel’ with its successor, the Cavendish subgroup of the triploid (AAA) banana cultivar group, which were resistant to *Foc* race 1 [12]. *Foc* race 1 affects banana cultivars such as ‘Gros Michel’ (AAA), ‘Maqueño’ (AAB), ‘Silk’ (AAB), ‘Pome’ (AAB), and ‘Pisang Awak’ (ABB) [14]. The emergence of *Foc* race 4 from Southeast Asia in the 1960s led to the decline of Cavendish bananas and other cultivated forms once again [15]. *Foc* race 4 is further divided into two groups: subtropical race 4 (STR4), which infects Cavendish under a cooler subtropical climate, and TR4, which causes infection in both tropical and subtropical climates [16,17].

*Foc* TR4 has spread globally, severely affecting banana plantations, and reached Latin America’s major banana-producing regions in 2019 [18]. It was first recorded in Southeast Asia in the early 1990s, and later in Australia’s Northern Territory in 1997 [19]. However, it was not until 2015 that *Foc* TR4 was detected in North Queensland, particularly in Tully, prompting a major increase in biosecurity measures and quarantine controls in the region [20].

FWB is a challenging disease to manage, making it crucial to minimise the spread of infection through quarantine measures and the use of disease-free propagative materials [21]. In regions where the disease has already spread, there are no effective chemical control options, so efforts have shifted towards developing banana cultivars with genetic resistance to *Foc* [22,23,24]. Somaclonal variants derived from Cavendish subgroup bananas have shown enhanced resistance to both *Foc* TR4 and *Foc* STR4 [15,25]. The resistance of various banana genotypes to these races has been evaluated in both field and pot trials [26,27,28,29]. Research suggests that rhizomes play a crucial role in a plant’s response to *Foc* [26]. Certain somaclonal variants, particularly the Giant Cavendish tissue culture variants (GCTCV), have demonstrated promising results in field trials in the Philippines [30], Australia [27], Africa [31], and China [28], as well as in pot trials [26,28].

Conventional crossing has been employed to develop *Foc* resistant banana cultivars. The Honduran Foundation for Agricultural Research (FHIA) initiated a banana breeding program aimed at creating resistant cultivars as a sustainable approach to combat various diseases, offering alternatives to the predominantly susceptible Cavendish bananas [32]. FHIA cultivars include synthetic hybrids (SH) of dwarf dessert bananas, plantains, and cooking bananas, some of which exhibit resistance to *Foc* race 4. Field evaluations have shown that some FHIA cultivars can be as productive and robust as natural hybrids [33]. Despite their high yields and resistance to FWB, these hybrids have not fully met market preferences [34]. The FHIA hybrids were developed from a set of diverse progenitor hybrids, which have been widely used in breeding programs at institutions such as the International Institute for Tropical Agriculture (IITA) in East and West Africa, the National Agricultural Research Organisation (NARO) in Uganda, the Brazilian Agricultural Research Corporation (EMBRAPA), and the Centre for International Cooperation in Agricultural Research (CIRAD) in France. FHIA lines have also been used to study disease responses against *Foc* TR4 in both field and pot trials [26,27].

Previous studies in many different host species have demonstrated a series of plant defence reactions against *Fusarium* pathogens, including papilla formation, production of antimicrobial substances, cell wall lignification, occlusion by gums, gels, or tyloses within xylem vessels, and vessel crushing [35,36]. The roles of each of these reactions in plant resistance are unclear, but they all contribute to the overall resistance capacity in plants. For instance, tyloses formation is considered a protective response of trees and herbaceous plants to vascular damage whether from mechanical injury or fungal or bacterial infections [37]. Tyloses formation occurs in the vessel lumens of root xylem in infected plants. They are formed from the extension of parenchyma cells through the pit membrane of the inner xylem wall and can fill up the entire xylem lumen. The amount of tyloses accumulation can vary depending on the resistance level of a cultivar. VanderMolen et al. (1987) showed that tyloses formation occurred in both susceptible and resistant cultivars, where rapid occlusion with tyloses occurred in infected root xylem vessels of resistant cultivars, whereas susceptible cultivars showed a similar tyloses initiation with plant growth reduced at later stages [38]. Tyloses formation has been shown to have a significant role in plant defence mechanisms in relation to the susceptibility of banana plants.

Green fluorescent protein (GFP)-tagged *Foc* strains have been used to track the movement of *Foc* in banana hosts resistant and susceptible to *Foc* TR4 [39,40] and *Foc* STR4 [11]. In the study presented here, the histological process of *Foc* infection was investigated using five banana cultivars: ‘Williams’ Cavendish (AAA), its somaclone ‘GCTCV119’ (AAA), as well as the Pome type cultivar known in Australia as ‘Lady Finger’ (AAB), ‘FHIA02’ (AAAA), and ‘FHIA25’ (AAB) hybrids. We observed that host susceptibility was linked to the active proliferation of the fungus within the xylem vessels of the rhizomes. In contrast, the fungus was restricted in the rhizomes of the *Foc* resistant cultivars like ‘FHIA25’ and ‘GCTCV119’. However, *Foc* was still able to enter the roots of these resistant cultivars, suggesting that resistance may not completely prevent initial fungal entry, but rather limit its spread. As *Foc* TR4-resistant cultivars are already being deployed in regions affected by the disease, understanding the true level of resistance is crucial. This includes examining how resistant cultivars respond to *Foc* infection, whether fungal presence is still detectable, and the nature of their resistance mechanisms. Addressing these questions is vital for assessing the effectiveness of deploying these resistant genotypes in regions affected by *Foc* TR4.

## 2. Materials and Methods

### 2.1. Plant Material

Tissue-culture banana plantlets of the *Foc* STR4 susceptible cultivars ‘FHIA02’, ‘Lady Finger’, ‘Williams’, and the *Foc* STR4 tolerant/resistant cultivars ‘GCTCV119’ (somaclonal variant of ‘Williams’) and ‘FHIA25’ were de-flasked into 30 cell potting trays (individual cells 5.8 cm × 5.8 cm × 5.5 cm). The plantlets were incubated on a lab bench at 20–22 °C under a 16/8 h day/night cycle of fluorescent light. The soil mix, UQ23, was steam-pasteurised, and contained 70% composted pine bark 0–5 mm in size and 30% coco peat, and had a pH range of 5.5–6.5. After 4 weeks of hardening off post-tissue culture, plants were repotted into 140 mm diameter pots (1.4 L in volume). A teaspoon of a balanced fertiliser (Osmocote) was added to each pot. The plants were moved to a temperature-controlled glasshouse with day and night temperatures maintained at 22 °C and 26 °C, respectively. They were grown for 6 weeks under a 16/8 h day/night photoperiod, supplemented with high-pressure sodium lamps. Watering was conducted to field capacity once every 2–3 days.

### 2.2. Fungal Strain and Inoculum Preparation

The GFP-tagged *Foc* STR4 (GFP-*Foc*-STR4) strain UQ6817 was derived from the strain BRIP40389 (Queensland Plant Pathology Herbarium) and has been described in previous studies with respect to its pathogenicity on *Musa* spp. [26,41]. The strain was single-spored and stored in the form of water-agar plugs in sterile water at 4 °C.

The GFP-*Foc*-STR4 strain was grown on full strength potato dextrose agar (PDA, Merck, Darmstadt, Germany), supplemented with 100 mg/L hygromycin B, and was incubated at 25 °C for 4 days. Four 5 mm^3^ mycelial blocks of GFP-*Foc*-STR4 were cut from a fully colonised PDA plate and were used to inoculate 500 mL of sterile potato dextrose broth (PDB, Merck, Darmstadt, Germany), supplemented with 50 mg/L hygromycin B. After four days of incubation at 28 °C, and on an orbital shaker at 180 rpm, the culture was filtered through four layers of sterile Miracloth (Merck, Rahway, NJ, USA). The spores were collected and washed with sterile distilled water (SDW).

### 2.3. Inoculations and Growth Conditions

For each selected cultivar, 30 plants with five to six healthy leaves and a 30 cm stem height were selected. The roots were washed with SDW and dipped for two hours in the GFP-*Foc*-STR4 spore suspension (2 × 10^6^ spores per mL). Soil from each pot was transferred into a clean disposable bag, and additional spore suspension (approximately 50,000 spores per gram of soil) was added and hand mixed. For the non-inoculated control, roots were dipped in SDW. All plants, GFP-*Foc*-STR4 treated and controls, were transplanted into 200 mm diameter (4 L volume) pots containing the UQ23 soil mix.

Plants subjected to scanning electron microscopy were inoculated with 45 g of GFP-*Foc*-STR4 infested Japanese millet (*Echinochloa esculenta*) variety ‘Shirohie’, as previously described [26]. A spore suspension of the same concentration and application method as above was also directly poured onto the root zone of the plants. Non-inoculated plants served as controls.

### 2.4. Symptoms Assessment and Reisolation

After inoculation with GFP-*Foc*-STR4, plants were harvested at weekly intervals for a total of 70 days during the experiment. At each collection time point, external symptoms were visually assessed on plant leaves and pseudostems. Internal symptoms, including discolouration within the roots, rhizome, and stems were assessed at 5–70 dpi when confocal microscopy was performed to detect the localisation of GFP-*Foc*-STR4 inside the host.

GFP-*Foc*-STR4 re-isolation was performed on ‘FHIA02’ and ‘FHIA25’. Plants were surface sterilised with 0.5% bleach (30 s), washed twice in SDW, and blotted dry on sterile paper towel under a laminar flow hood. Sections (2–5 mm) of leaf, petiole, and stem just above the rhizome, the central cylinder of the rhizome, and the rhizome node connecting roots were cut and embedded into water agar. After 10 days of incubation at 25 °C in the dark, the *Fusarium*-like colonies (white mycelia with pink to mauve staining of medium producing macro- and microconidia) were sub-cultured onto half strength PDA supplemented with 100 mg/L hygromycin B at 25 °C in the dark for 10 days and examined under a confocal microscope to confirm GFP fluorescence.

### 2.5. Investigation of Fungal Colonisation

The colonisation of inoculated banana plants by GFP-*Foc*-STR4 was monitored at 5–70 dpi using laser scanning and scanning electron microscopies. 

#### 2.5.1. Laser Scanning Microscopy

For visualisation under a confocal microscope, plant tissues, including roots, stems, rhizomes, and leaf parts, were sectioned transversely and longitudinally using a double-edged razor blade. The tissue sections were mounted in sterile deionised water for imaging.

GFP-*Foc*-STR4 was detected using a Zeiss 700 laser scanning microscope (Zeiss, Oberkochen, Germany) and a laser at an excitation wavelength of 488 nm. The Z-stack function was used to capture 3D images consisting of 10–30 optical slices taken at intervals of 1–5 μm. The T-PMT (transmission detector setting) was also used to view the sectioned plant tissues in an overlay of brightfield.

#### 2.5.2. Scanning Electron Microscopy

At 2- and 6-weeks post-inoculation, primary roots and rhizomes were collected and prepared for observation under a HitachiTM4000Plus Bench top (Hitachi High-Tech, Tokyo, Japan) scanning electron microscope (SEM) in a high-vacuum mode operating at 15 kV and a working distance of 15–18 mm.

The sample preparation protocol was adapted from Ratnayake et al. [42]. In brief, samples (0.5–1 cm) were immediately fixed in 2.5% (*v*/*v*) glutaraldehyde in 0.1 M sodium phosphate buffer (pH 6.8). The samples, embedded in agarose (50%, *w*/*v*), were sliced at 100 μm with a vibratome (VT1000 S, Leica Biosystems, Wetzlar, Germany). Sections were stored in 6% sodium azide buffer. 

Excised samples were washed twice in 0.1 M sodium phosphate buffer (pH 6.8) to remove the glutaraldehyde, and processed in a Biowave microwave (Ted Pella, Redding, CA, USA) operating at 150 W per 1 min, following 1 min off and further 1 min treatment. Then, the samples were dehydrated in a graded (60, 70, 80, 90, and two 100%) series of ethanol, and dried in hexamethyldisilazane (HMDS)/absolute ethanol solution (1:1, *v*/*v*), and twice in absolute ethanol. Samples were left overnight to evaporate HMDS. 

Each dried section was mounted onto aluminium stub using double-sided sticky carbon tabs (ProSciTech, Kirwan, Australia), and sputter coated with platinum (EIKO IB-5 Sputter Coater, EIKO Engineering Co., Ltd., Hitachinaka, Japan), ensuring a complete and uniform film (∼15 nm thick) over the surfaces. Approximately 400 sections were imaged. The presence/absence of tyloses within the tissue due to GFP-*Foc*-STR4 inoculation was recorded and compared with findings from similar studies [38,43].

## 3. Results

### 3.1. Symptoms Assessment and Reisolation

**Figure 1:**
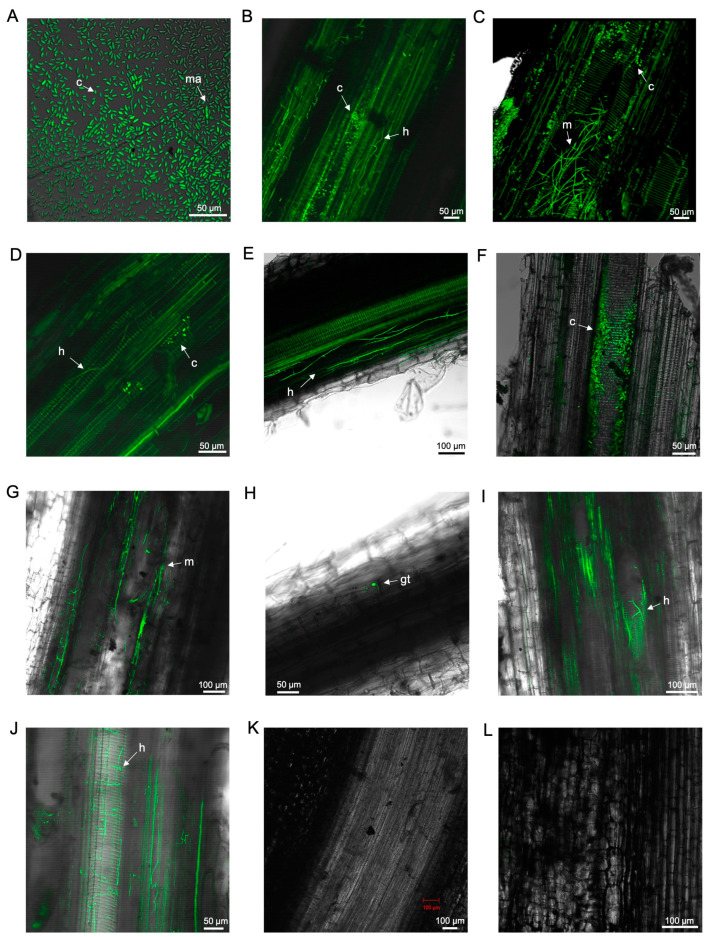
Laser scanning microscopy pictures: (**A**) GFP-*Foc*-STR4 suspension of macroconidia and microconidia. Localisation of GFP-*Foc*-STR4 in banana cultivars during the infection process: (**B**) Mycelia and microconidia within the xylem of a lateral root in ‘FHIA02’ at 5 dpi. (**C**) Microconidia and mycelia between the cortical cells of the elongation zone and the xylem vessels of the primary root in ‘FHIA02’ at 5dpi. (**D**) Microconidia and hyphae visualised in the elongation zone of a ‘FHIA25’ lateral root at 5 dpi. (**E**) Hyphae along the epidermis of a ‘Williams’ lateral root at 5 dpi. (**F**) Microconidia proliferating in the xylem vessels of a ‘Williams’ lateral root at 9 dpi. (**G**) Mycelial networks in the xylem vessels of a ‘GCTCV119’ lateral root at 5 dpi. (**H**) Germ tube on the epidermis of a ‘GCTCV119’ lateral root at 7 dpi. (**I**) Hyphae within the vasculature of a ‘GCTCV119’ lateral root at 7 dpi. (**J**) Hyphae in the xylem vessels of a ‘Lady Finger’ lateral root at 8 dpi. Longitudinal section of non-inoculated lateral root in ‘FHIA02’ at 5 dpi (**K**) and ‘FHIA25’ at 6 dpi (**L**). Abbreviations are annotated as: ma = macroconidia; c = conidia; h = hyphae; m = mycelium; gt = germ tube. Horizontal bars indicate the scale used to capture the images.

Under the confocal microscope, the GFP-*Foc*-STR4 suspension showed strong fluorescence in both microconidia and macroconidia, attributed to the constitutive expression of GFP (Figure 1A).

Symptoms assessment at 32–35 dpi revealed varying degrees of internal discoloration in the rhizomes of all GFP-*Foc*-STR4 inoculated plants, except ‘GCTCV119’ (Figure S1). Extensive discoloration was also observed in the pseudostems of all tested cultivars. *F. oxysporum*-like colonies were reisolated from the rhizomes and lower stems of ‘FHIA02’ and ‘FHIA25’ plants at 35 dpi, and the presence of GFP-*Foc*-STR4 was confirmed (Table S1).

### 3.2. Laser Scanning Microscopy

#### 3.2.1. Observations on Susceptible Cultivar FHIA02

At 5 dpi, highly abundant microconidia and mycelial networks were detected under a confocal microscope in the xylems of both the lateral and main roots of ‘FHIA02’ plants (Figure 1B,C).

**Figure 2:**
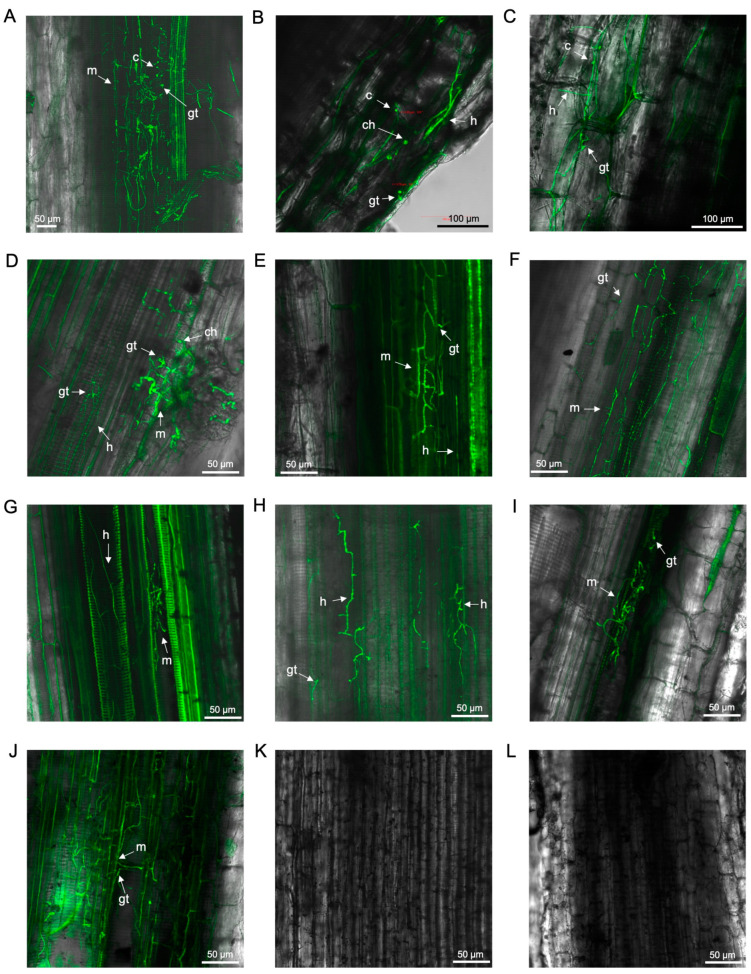
Laser scanning microscopy showing the localisation of GFP-*Foc*-STR4 in ‘FHIA02’ during the infection process: (**A**) Microconidia and mycelia colonising the xylem vessels of a fine root at 12 dpi. (**B**) Germ tube, hyphae, and chlamydospores in the epidermis of a lateral root at 14 dpi. (**C**) Germ tube, hyphae, and chlamydospores in the cortex region of a lateral root at 14 dpi. (**D**) A wound site penetrated by GFP-*Foc*-STR4 on a lateral root at 19 dpi. (**E**) Germ tubes and mycelia in the xylem vessels of a fine root at 26 dpi. (**F**) Germ tubes and mycelia in the xylem vessels of a primary root at 29 dpi. (**G**) Mycelia within the xylem vessels of a primary root near a rhizome node at 36 dpi. (**H**) Germ tubes and hyphae in the cortex of a main root at 41 dpi. (**I**) Germ tubes and mycelia in the xylem vessels of the rhizome connecting the main root at 41 dpi. (**J**) Germ tubes and mycelia colonising the xylem vessels of the rhizome connecting the main root at 42 dpi. Non-inoculated control of a fine root at 18 (**K**) and 62 (**L**) dpi. Abbreviations are annotated as: ch = chlamydospores; c = conidia; h = hyphae; m = mycelium; gt = germ tube. Horizontal bars indicate the scale used to capture the images.

Between 12 and 70 dpi, it was evident that the fungus moved uninhibited through the plant vasculature, affecting both the roots and rhizomes of ‘FHIA02’ (Figure 2). At 12 dpi, extensive mycelial networks were detected in the fine roots (Figure 2A). By 14 dpi, chlamydospores and a spore germ tube were visible on the epidermis of lateral roots (Figure 2B), and intercellular hyphal movement was observed (Figure 2C). At 19 dpi, the fungus appeared to establish itself through a wound site in a root hair (Figure 2D). From 26 to 42 dpi, extensive mycelial networks and germinated spores were found not only in the fine roots but also in the main roots and root nodes, which connected to the rhizome (Figure 2E–J).

**Figure 3:**
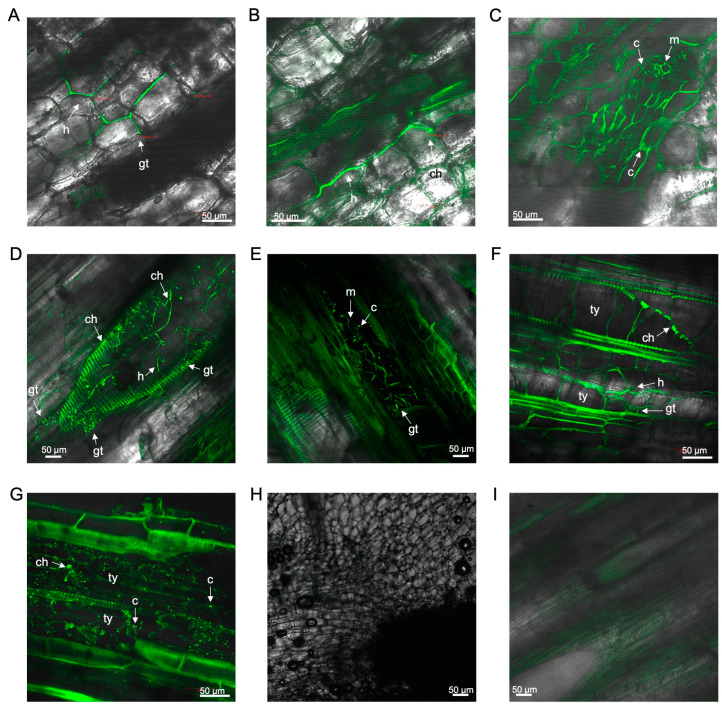
Laser scanning microscopy showing the localisation of GFP-*Foc*-STR4 in the rhizome of ‘FHIA02’ during the infection process. (**A**) Germ tubes and hyphae in the cortical cells of a discoloured rhizome at 14 dpi. (**B**) Hyphae and chlamydospores in a discoloured region of a rhizome at 14 dpi. (**C**) Microconidia and mycelial networks in the rhizome at 14 dpi. (**D**) Germ tubes, hyphae, and chlamydospores in the vascular vessel of a rhizome at 36 dpi. (**E**) Germ tubes, microconidia, and mycelia in the xylem vessel of a rhizome at 36 dpi. (**F**) Tyloses, germ tubes, hyphae, and chlamydospores in the xylem vessel lumen of a rhizome node at 68 dpi. (**G**) Tyloses, microconidia, and chlamydospores in the xylem vessels of a rhizome at 70 dpi. Non-inoculated control of a rhizome at 14 dpi (**H**) and 68 dpi (**I**). Abbreviations are annotated as: ch = chlamydospores; c = conidia; h = hyphae; m = mycelium; gt = germ tube; ty = tyloses. Horizontal bars indicate the scale used to capture the images.

In the rhizome of ‘FHIA02’, the intercellular presence of the fungus through the rhizome cortex was observed as early as 14 days dpi (Figure 3A–C). An intercellular hypha with a single terminal chlamydospore was also visualised (Figure 3B). Strong vascular autofluorescence, with microconidia forming in clusters, was detected in the rhizome at 14 dpi (Figure 3C). By 36 dpi, abundant germinated microconidia were detected in the xylem vessels (Figure 3D,E). Tyloses were noted as fine angular intra-xylem walled networks, resembling those described by VanderMolen et al. [38], which auto-fluoresced along with the plant cell wall structures of the xylem (Figure 3D,E). These tyloses appeared to occlude vessels in the rhizome at 68–70 dpi (Figure 3F,G). Microconidia and chlamydospores were co-localised in and around the xylem vessels (Figure 3D,E,G).

**Figure 4:**
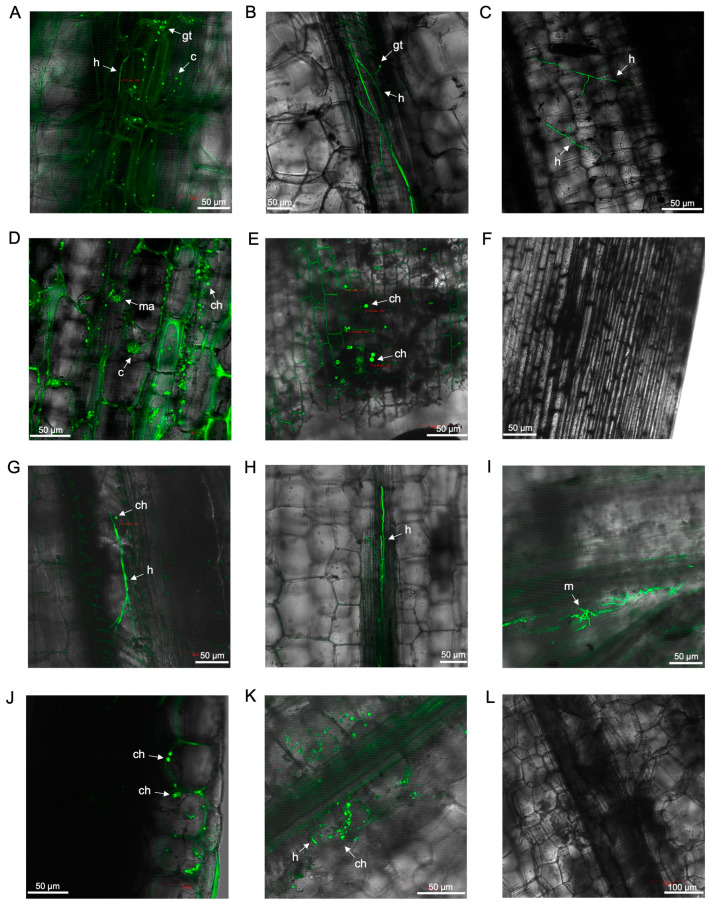
Laser scanning microscopy showing the localisation of GFP-*Foc*-STR4 in the leaves of ‘FHIA02’ and ‘FHIA25’. (**A**) Germ tubes, microconidia, and hyphae on an outer leaf sheath of ‘FHIA02’ at 14 dpi. (**B**) Germ tube and hyphae visualised in the xylem vessel of an outer leaf sheath of ‘FHIA02’ at 26 dpi. (**C**) Hyphae at the epidermis of an outer leaf sheath of ‘FHIA02’ at 29 dpi. (**D**) Microconidia, hyphae, and chlamydospores at the edge of a petiole of a senescing leaf in ‘FHIA02’ at 54 dpi. (**E**) Chlamydospores at the edge of a petiole of a senescing leaf in ‘FHIA02’ at 62 dpi. (**F**) Non-inoculated control at the edge of a petiole in ‘FHIA02’ at 63 dpi. (**G**) Hyphae and chlamydospore confined to the xylem vessel in the outer leaf sheath of ‘FHIA25’ at 16 dpi. (**H**) Hyphae confined to the xylem vessel in the outer leaf sheath of ‘FHIA25’ at 21 dpi. (**I**) Mycelial networks in the xylem vessels of a leaf of ‘FHIA25’ at 29 dpi. (**J**) Chlamydospores in a discoloured region at the edge of a petiole of ‘FHIA25’ at the 62 dpi. (**K**) Chlamydospores and hyphae at the edge of a petiole in ‘FHIA25’ at 70 dpi. (**L**) Non-inoculated control in the midrib of a ‘FHIA25’ leaf at 70 dpi. Abbreviations are annotated as: ma = macroconidia; ch = chlamydospores; c = conidia; h = hyphae; m = mycelium; gt = germ tube. Horizontal bars indicate the scale used to capture the images.

The presence of GFP-*Foc*-STR4 was detected in the outer leaf sheath and petiole edge of ‘FHIA02’ plants (Figure 4). Microconidia and hyphae were detected as early as 14 dpi (Figure 4A). The fungus was confined to the xylem vessels in the midrib of the leaf sheath at 26 dpi (Figure 4B), with mycelial networks observed on the epidermis of leaf sheaths at 29 dpi (Figure 4C). At 54–62 dpi, macroconidia and chlamydospores were abundantly visible along the petiole edge of a senescing leaf in ‘FHIA02’ (Figure 4D,E).

#### 3.2.2. Observations on Resistant Cultivar FHIA25

The presence of GFP-*Foc*-STR4 in the tolerant/resistant cultivar ‘FHIA25’ was detected less frequently than in ‘FHIA02’. This was especially evident at 5 dpi, where only a single hyphal strand and a few conidia were observed (Figure 1D).

**Figure 5:**
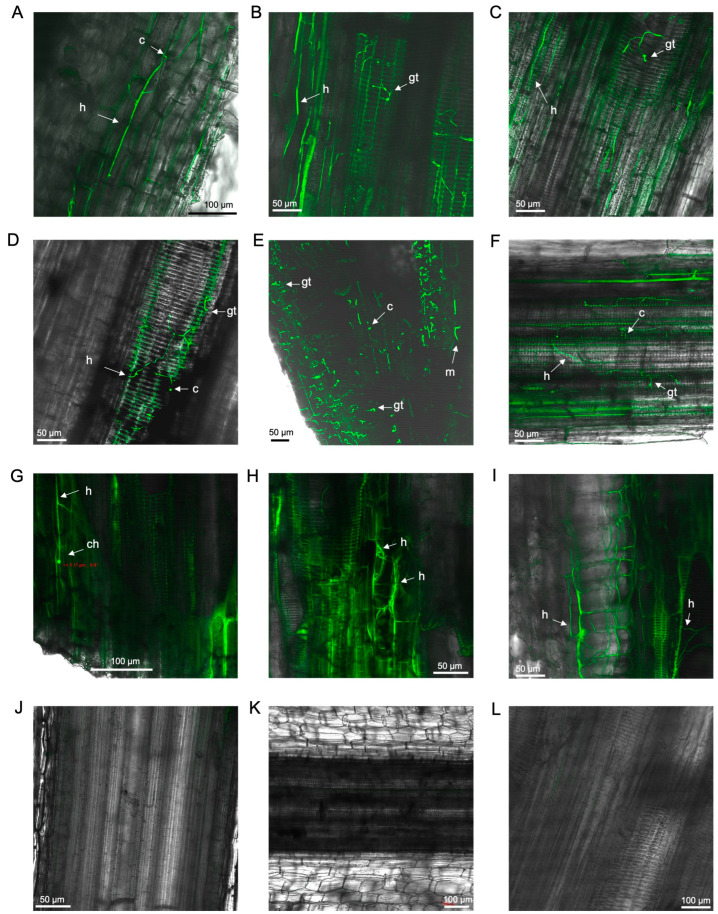
Laser scanning microscopy showing the localisation of GFP-*Foc*-STR4 in the roots of ‘FHIA25’ during the infection process. (**A**) Microconidia and hyphae visualised at the epidermis of a fine root at 12 dpi. (**B**) Germ tubes and hyphae in the xylem vessel of a fine root at 12 dpi. (**C**) Germ tubes and hyphae in the xylem vessel of a fine root at 21 dpi. (**D**) Germ tubes, hyphae, and microconidia in the xylem vessel of a junction between lateral root and rhizome at 26 dpi. (**E**) Germ tubes, microconidia, and mycelia at the epidermis of a primary root tip at 29 dpi. (**F**) Germ tubes, microconidia, and hyphae in the xylem vessel of a fine root at 29 dpi. (**G**) Hyphae and a single terminal chlamydospore in the xylem vessel of a fine root at 36 dpi. (**H**) Hyphae and GFP autofluorescence in the xylem of a junction between root and rhizome at 49 dpi. (**I**) Hyphae and GFP autofluorescence in the xylem of a junction between root and rhizome at 49 dpi. (**J**) Non-inoculated control in a lateral root at 21 dpi (**J**) and 36 dpi (**K**). (**L**) Non-inoculated control in the junction between lateral root to rhizome at 51 dpi. Abbreviations are annotated as: ch = chlamydospores; c = conidia; h = hyphae; m = mycelium; gt = germ tube. Horizontal bars indicate the scale used to capture the images.

The presence of GFP-*Foc*-STR4 in the fine roots was clearly visualised from 12 to 49 dpi (Figure 5). The extent of root colonisation by the fungus appeared similar to that in ‘FHIA02’. At 12 dpi, microconidia on false heads in monophialides were observed in the fine roots (Figure 5A), and germinated spores were detected in the xylem vessels at 14 dpi (Figure 5B). The infection continued to spread, with clear fungal colonisation of xylem vessels by 21 dpi (Figure 5C) and extension through lateral root nodes connecting to the rhizome by 26 dpi (Figure 5D). This consistent germination of spores persisted at later time points up to 29 dpi, with germinated microconidia and hyphae visible in the root cap and elongation zone (Figure 5E,F).

Plants often respond to xylem-invading vascular wilt pathogens by depositing vascular coatings of lignin and suberin in colonised vessels [44]. Vascular coatings, such as lignin and suberin, are visualised as GFP autofluorescence from phenolic deposits in vessels colonised by vascular wilt pathogens [44]. Strong GFP fluorescence was observed in colonised compartments from 36 to 49 dpi, suggesting the presence of vascular coatings (Figure 5G–I). The GFP autofluorescence appeared co-localised with the fungus in these regions, indicating an active defence response.

**Figure 6:**
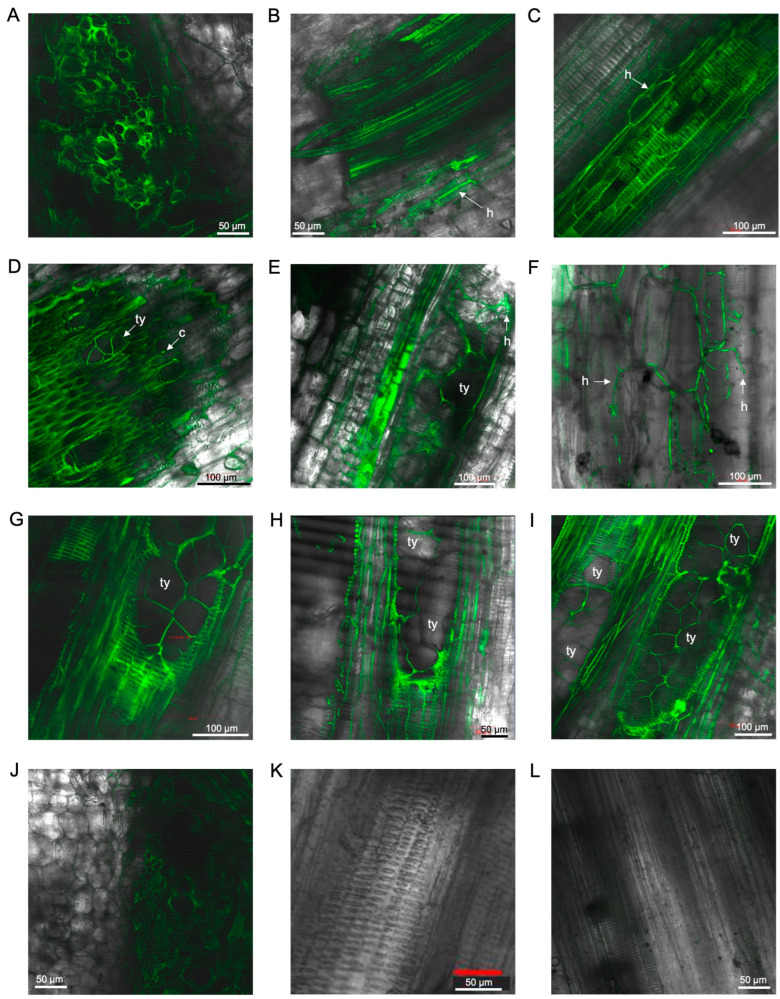
Laser scanning microscopy showing the localisation of GFP-*Foc*-STR4 in the rhizome of ‘FHIA25’ during the infection process. (**A**) GFP autofluorescence in the rhizome at 14 dpi. (**B**) Hyphae and GFP autofluorescence in the xylem vessels of a rhizome at 14 dpi. (**C**) Hyphae and GFP autofluorescence in the xylem of a junction between root and rhizome at 36 dpi. (**D**) Tyloses and microconidia in the vascular bundles of a rhizome at 36 dpi. (**E**) Tyloses and hyphae in the xylem vessel of a junction between root and rhizome at 41 dpi. (**F**) Hyphae in a junction between primary root and rhizome at 41 dpi. (**G**) Tyloses in the xylem of a junction between primary root and rhizome at 42 dpi. (**H**) Tyloses in the xylem vessel of a junction between primary root and rhizome at 62 dpi. (**I**) Tyloses in xylem vessels of a junction between primary root and rhizome at 70 dpi. (**J**) Non-inoculated control in the rhizomes at 16 dpi (**J**), 36 dpi (**K**), and 70 dpi (**L**). Abbreviations are annotated as: c = conidia; h = hyphae; ty = tyloses. Horizontal bars indicate the scale used to capture the images.

In the rhizome of ‘FHIA25’, the presence of Foc STR4 appeared more restricted (Figure 6). Strong GFP autofluorescence marked infected regions in the rhizome and the main root nodes connecting to the rhizome (Figure 6A,D,E,G–I). The GFP-Foc-STR4 presence was primarily confined to the xylem vessels (Figure 6B,C). At 36–41 dpi, tyloses were observed in the vasculature of the rhizome (Figure 6D,E), with hyphal movement restricted between individual tyloses (Figure 6F). Tyloses formation continued to be detected in the rhizome from 42 to 70 dpi (Figure 6G–I), and fungal presence in these regions remained minimal, suggesting a more effective containment in ‘FHIA25’.

The presence of GFP-*Foc*-STR4 was detected in the outer leaf sheath and petiole edge of ‘FHIA25’ plants (Figure 4). Microconidia and hyphae were detected as early as 16 dpi (Figure 4G). By 21 dpi, the fungus was confined to the xylem vessels in the midrib of the leaf sheath (Figure 4H), with mycelial networks observed on the epidermis of leaf sheaths at 29 dpi (Figure 4I). At 62–70 dpi, chlamydospores and hyphae were abundantly observed in the petiole of ‘FHIA25’ (Figure 4J,K).

#### 3.2.3. Observations on Cultivars Williams, GCTCV119 and Lady Finger

In ‘Williams’, extensive microconidia and hyphae were detected in the lateral roots at 5 dpi (Figure 1E) and appeared to occlude the xylem in these roots by 9 dpi (Figure 1F). Similarly, abundant mycelia were observed in the xylem vessels of the lateral roots in ‘Lady Finger’ plants at 8 dpi (Figure 1J). In ‘GCTCV119’ plants, GFP-*Foc*-STR4 hyphae were present in the lateral roots at 5–7 dpi (Figure 1G,I) but to a lesser extent than in ‘Williams’, ‘Lady Finger’, or ‘FHIA02’.

GFP-*Foc*-STR4 chlamydospores and hyphae were visualized in the vasculature of primary roots of ‘GCTCV119’, ‘Williams’, and ‘Lady Finger’ from 15 to 45 dpi (Figure S2).

To assess the movement from the roots into the rhizome, the presence of GFP-*Foc*-STR4 was checked in the connection between root nodes and corms at 32–60 dpi. GFP-*Foc*-STR4 hyphae and mycelia were present in the rhizome nodes of all three cultivars, indicating movement through the vasculature from the roots into the rhizome (Figure S3).

In the rhizomes, however, GFP-*Foc*-STR4 was not detected in ‘GCTCV119’ 26–42 dpi (Figure S4A–C). In contrast, chlamydospores and hyphae were clearly present in the vasculature of the rhizomes in ‘Lady Finger’ and ‘Williams’ at 27–40 dpi (Figure S4D–I), with strong GFP fluorescence observed in the infected regions.

**Figure 7:**
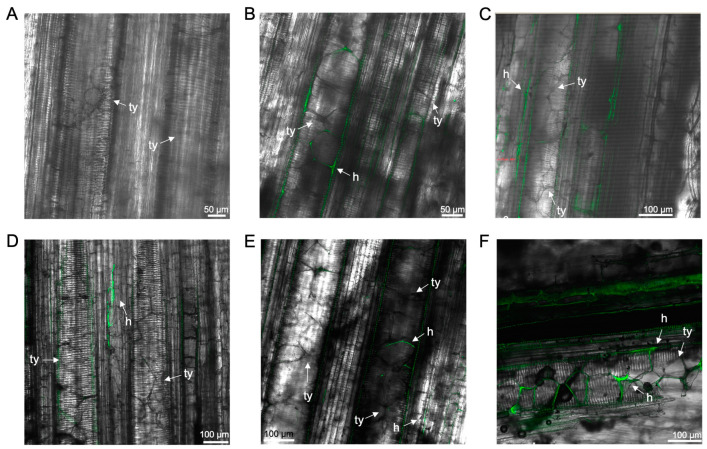
Laser scanning microscopy showing tyloses formation and the localisation of GFP-*Foc*-STR4 in the roots and rhizome of ‘GCTCV119’ and ‘Williams’. (**A**) Tyloses in the xylem vessel of a non-inoculated ‘GCTCV119’ plant at 56 dpi. (**B**) Tyloses and hyphae in the main root of ‘GCTCV119’ at 56 dpi. (**C**) Tyloses and hyphae in the xylem vessel of a rhizome of ‘GCTCV119’ at 59 dpi. (**D**) Hyphae confined in between two occluded vessels in the primary root of ‘Williams’ at 56 dpi. (**E**) Tyloses and hyphae in the xylem vessel of the main root of ‘Williams’ at 56 dpi. (**F**) Tyloses and hyphae in the xylem vessel of a rhizome of ‘Williams’ at 55 dpi. Abbreviations are annotated as: h = hyphae; ty = tyloses. Horizontal bars indicate the scale used to capture the images.

Tyloses were observed within the vascular bundles of primary roots and the rhizome in ‘GCTCV119’ and ‘Williams’ plants at 55–59 dpi (Figure 7). In ‘GCTCV119’, tyloses were also formed in the non-inoculated controls, suggesting that tyloses formation can occur naturally in plants (Figure 7A). In both ‘Williams’ and its somaclonal derivative ‘GCTCV119’, GFP-*Foc*-STR4 appeared confined within occluded xylem vessels (Figure 7B,E,F) or located between two adjacent vessels (Figure 7D).

Above ground, GFP-*Foc*-STR4 was detected in the pseudostem of ‘Lady Finger’ and ‘Williams’ at 18–20 dpi (Figure S5A–C), but was not in ‘GCTCV119’ at 28 dpi (Figure S5E). At 51–59 dpi, GFP-*Foc*-STR4 mycelia were found in the pseudostem of all three cultivars (Figure S5B,D–F). In the leaves of all three cultivars presented in this section, mycelial networks and sporodochia were consistently present at both early (13–40 dpi) and late (60–65 dpi) stages (Figure S5G–L).

### 3.3. Scanning Electron Microscopy

**Figure 8:**
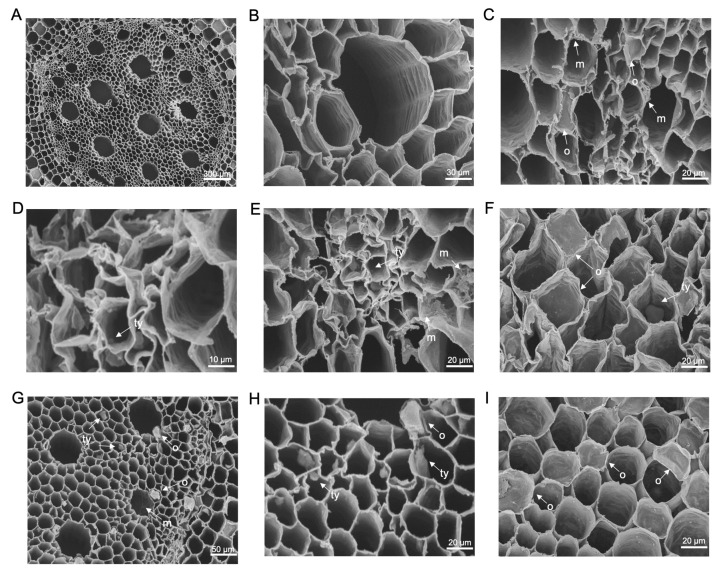
Scanning electron micrographs showing transverse sections of primary roots from ‘FHIA02’. A non-inoculated control of a root (**A**) and under magnified view (**B**) at 14 dpi. (**C**) Mycelia and vascular occlusion in the vascular cavities of the main root at 14 dpi. (**D**) Tyloses in the vascular cavities of the main root at 14 dpi. (**E**) Tyloses and mycelia in the vascular cavities of the main root at 14 dpi. (**F**) Tyloses and vascular occlusion in the vascular cavities of the main root at 14 dpi. (**G**,**H**) Tyloses and vascular occlusion in the xylem cavities of the main root at 42 dpi. (**I**) Tyloses occluding multiple xylem cavities in the main root at 42 dpi. Abbreviations are annotated as: m = mycelium; ty = tyloses; o = vascular occlusion. Horizontal bars indicate the scale used to capture the images.

**Figure 9:**
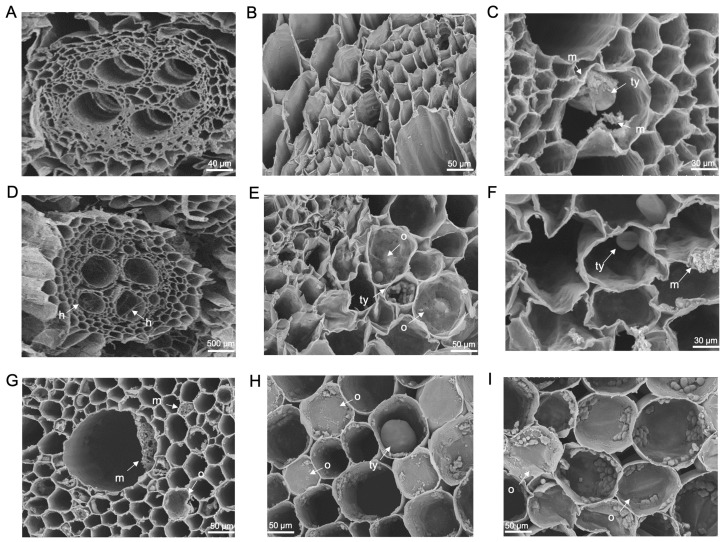
Scanning electron micrographs showing transverse sections of primary roots from ‘FHIA25’. Vascular bundles in the primary root of a non-inoculated plant (**A**) and under a magnified view (**B**) at 14 dpi. (**C**) Tyloses formation and mycelia in the same xylem cavity of a primary root at 14 dpi. (**D**) Hyphae in the xylem cavities of the primary root at 14 dpi. (**E**) Tyloses formation and vascular occlusion in the xylem cavities of the primary root at 14 dpi. (**F**) Tyloses formation, vascular occlusion, and mycelia in the xylem cavities of the primary root at 14 dpi. (**G**) Mycelial networks in the xylem cavities of the primary root at 42 dpi. (**H**,**I**) Tyloses formation and vascular occlusion in the xylem cavities of the primary roots at 42 dpi. Abbreviations are annotated as: m = mycelium; ty = tyloses; o = vascular occlusion. Horizontal bars indicate the scale used to capture the images.

SEM analysis of the primary roots of both ‘FHIA02‘ and ‘FHIA25‘ detected the presence of tyloses and fungal structures within xylem vessels (Figures 8 and Figure 9). Tyloses were clearly visible in the vascular bundles of both cultivars at 14 and 42 dpi (Figure 8C,E,F,H,I and Figure 9C,F,H,I). Various sizes of tyloses, including those in the process of forming, were observed in the xylem vessels of plants inoculated with GFP-*Foc*-STR4 (Figure 8F and Figure 9F,H). These tyloses were in close proximity to GFP-*Foc*-STR4 (Figure 8C) and, in some instances, were forming within the same vascular bundle as the fungal structures (Figure 9C). Additionally, tyloses were observed in the vascular bundles of control ‘FHIA02’ plants not treated with *Foc*, suggesting that tyloses can form during regular plant growth (Figure 8A).

Fungal growth was observed in the parenchyma cells and vascular bundles of both ‘FHIA02’ and ‘FHIA25’ at 14 and 42 dpi, aligning with GFP study findings that confirmed the presence of the fungus within the host at these time points. GFP-*Foc*-STR4 mycelia were clearly detected within individual vascular bundles and the pits of the main roots (Figure 8C,E and Figure 9C,G). In terms of root response, there were no significant differences between ‘FHIA02’ and ‘FHIA25’ in the number of tyloses formed or the extent of fungal colonization within the vasculature.

## 4. Discussion

Green fluorescent proteins have proven to be an invaluable tool for studying pathogen–host interactions in plants, allowing for real-time visualisation of fungal movement and colonisation within plant tissues [26,45,46]. In *F. oxysporum*, the production of microconidia and macroconidia is essential for the fungus to successfully proliferate within host plants. By tracking the accumulation of spores in specific plant compartments, researchers can gain insights into the dynamics of infection, which is crucial for managing and containing *Foc* in agricultural settings. However, the plant defence mechanisms in resistant or tolerant cultivars, such as ‘FHIA25’ and ‘GCTCV119’, are not yet fully understood, and these cultivars offer an important opportunity to explore how the host responds to pathogen attack.

The movement of *Foc* through the root systems was observed across all five banana cultivars, ‘Williams’, ‘Lady Finger’, ‘FHIA02’, ‘FHIA25’, and ‘GCTCV119’. Despite ‘FHIA25’ showing good resistance to *Foc* STR4, chlamydospores and microconidia from the GFP-*Foc*-STR4 isolate were found to attach to and germinate on the root tips and fine root hairs of this cultivar. Following this, the fungus was able to penetrate the root surface and spread through the root vascular system. This suggests that, while resistance mechanisms are present in certain cultivars, *Foc* can still actively enter banana hosts through their root systems.

In both ‘FHIA02’ and ‘FHIA25’, the fungus exhibited intercellular movement, initially in the epidermis and then through the elongation zones of the roots. This pattern aligns with the behaviour of biotrophic pathogens, similar to other *Fusarium* species, which rely on living plant cells to progress through the host [47,48]. The consistent detection of the fungus in the xylem vessels of both the roots and root nodes connecting to the rhizomes further suggests that vascular tissues play a critical role in facilitating the movement of the pathogen within the plant, regardless of the cultivar’s resistance level.

In the current study, a differential plant response to GFP-*Foc*-STR4 infection was observed in the rhizome tissues of the ‘FHIA02’ and ‘FHIA25’ banana cultivars. In ‘FHIA02’, a susceptible cultivar, proliferation of spores and mycelia was detected extensively in the xylem vessels of the symptomatic rhizomes. This indicates that the fungus is able to spread freely in the rhizome tissue, which likely contributes to the continued infection of the plant. In contrast, fewer hyphae were observed in the rhizome of the resistant cultivar ‘FHIA25’, and those present appeared to be confined to the xylem vessels, suggesting that the plant was mounting a defence response to restrict fungal spread. The formation of tyloses—cellular obstructions that block fungal movement—was detected in both cultivars, but was more pronounced in the resistant ‘FHIA25’, where it seemed to play a role in restricting the pathogen. Additionally, in the highly susceptible cultivars ‘Lady Finger’ and ‘Williams’, the rhizomes were heavily colonized by the fungus, further supporting their susceptibility to *Foc*. Meanwhile, the rhizomes of ‘GCTCV119’, a more tolerant cultivar, displayed limited fungal movement in the xylem vessels. This suggests that while the fungus can initially enter the plant, its spread in the rhizome is limited by the host plant’s defence mechanisms. Taken together, these observations emphasize the critical role of the rhizome in the banana–Fusarium interaction [26]. The plant’s ability to contain the fungus within the rhizome, possibly through mechanisms like tyloses formation, appears to be a key factor in determining resistance or susceptibility to *Foc*.

A similar pattern of restricted colonisation in *Dianthus caryophyllus* by *F. oxysporum* f. sp. *dianthi* has been reported, and further characterisation revealed that the infected regions of the xylem became compartmentalised by cell wall thickening, hyperplasia of parenchyma cells, and the built-up of vascular occluded materials [36]. In this study, vascular occlusion in the rhizome of ‘FHIA25’ was detected at 36 dpi and in ‘FHIA2’ at 68 dpi. SEM revealed that vascular occlusion in the main roots of both ‘FHIA2’ and ‘FHIA25’ occurred as early as 14 dpi following fungal inoculation. Similar occlusion was observed in the ‘GCTCV119’ and ‘Williams’ cultivars at 56–59 dpi. These results collectively suggest that vascular occlusion is an inducible plant defence mechanism to prevent the spread of an invading pathogen. While it may contribute to deterring the movement of the pathogen inside the host, tyloses formation [38] do not fully explain the host resistances observed in ‘FHIA25’ and ‘GCTCV119’. Formation of tyloses is typically triggered by infections [49], wounding [50], heartwood formation [51], and abscission [52]. The ability to form tyloses was found to be an important factor in resistance to *F. oxysporum* in cotton [53] and has been shown to be upregulated by an exogenous chemical application in banana [54]. Therefore, the presence of tyloses observed in this study is consistent with their roles in growth and development, as well as in the regulation of stresses including pathogen attacks and the activation of oxidative stress.

Another aspect of pathogen deterrence is the formation of vascular coatings in or around infected regions. These regions have been identified as plant physico-chemical barriers induced against xylem vascular wilt pathogens [44]. Particularly, gel and lignin depositions have been found to be associated with vertical and horizontal restrictions, respectively, of *Foc* in banana hosts [38,55]. These mechanisms, involving the formation of gels, gums, or mucilage in and around the vascular systems, have been shown to limit fungal growth in banana [43], tomato [56], carnation [57], pea [35,58], cotton [45,53], and bean plants [59]. These barriers are mostly composed of carbohydrates like pectin, polyphenols, and sometimes phytoalexins, lignin-like compounds, or lipoidal substances [35]. Upon a pathogen attack, it was observed in peas that the production of carbohydrates and polyphenolics by vascular parenchyma cells progressively accumulated in the lumen of xylem cells, highlighting important functions of these compounds in defence against *F. oxysporum* f. sp. *pisi* [35]. In this study, the movement of *Foc* through the aerial parts of banana plants was observed. Chlamydospores and hyphae were detected on the petiole and outer leaf sheath of both ‘FHIA02’ and ‘FHIA25’ plants, indicating that the fungus can penetrate and travel through the leaf sheaths. Although ‘FHIA25’ is resistant to *Foc* STR4, the fungus was still able to move through these tissues and reach the aerial parts of the plant. This observation highlights that while resistance mechanisms in the roots and rhizomes may limit the spread of the fungus, the pathogen can still access and potentially colonise the upper parts of the plant.

Additionally, sporodochia, which are fungal structures that produce conidia, were observed around the stomata in the leaves of both ‘Williams’ and ‘Lady Finger’. This suggests that the fungus can not only invade through the vascular tissue but may also spread externally, likely through the stomatal pores in the leaves, a known pathway for fungal pathogens. These findings align with previous studies showing the movement of *Foc* through the leaf sheaths [40] and its potential entry via stomata [11]. However, this study adds novel insight by reporting the transmission of *Foc* through the aerial parts of a resistant cultivar, *FHIA25*, which has not been documented before. This new observation underscores the complex nature of host–pathogen interactions and suggests that even resistant plants may not be fully immune to infection in all tissues.

This study highlights an important aspect of the ongoing battle against *Foc* TR4, as it shows that even cultivars considered resistant or tolerant to *Foc* TR4, such as ‘GCTCV119’ and ‘FHIA25’, can still be colonized by the fungus, though at varying levels of severity. While ‘GCTCV119’ and its improved variant, ‘GCTCV218’ (also known as ‘Formosana’), are acknowledged for their tolerance to *Foc* TR4, they are not fully immune [18,27,31], and their resistance can be compromised depending on the inoculum threshold and environmental conditions. The resistance of ’FHIA25’ to both *Foc* STR4 and *Foc* TR4 is well-documented [26,27], but this study suggests that the pathogen can still invade the plant tissues, albeit at lower frequencies compared to more susceptible cultivars like ‘FHIA02’ and ‘Williams’. This raises the question of how much inoculum these cultivars can tolerate before they succumb to the disease.

To make informed decisions about deploying these cultivars in regions affected by *Foc* TR4, further research is needed to establish the inoculum threshold levels that resistant or tolerant cultivars can withstand. Stress testing these cultivars under various field conditions will be crucial to understanding the limits of their resistance and optimizing their use in affected regions. This research will also guide the development of effective management strategies for controlling *Foc* TR4, ensuring that resistant cultivars can be effectively utilised in the field without prematurely succumbing to the disease.

## 5. Conclusions

This study examined the movement of a GFP-tagged *Foc* STR4 strain across five banana cultivars. Findings showed that in resistant cultivars, *Foc* was contained within the rhizome, while it spread further in susceptible cultivars. This suggests that the rhizome plays a critical role in limiting fungal spread within resistant plants. However, *Foc* was still detected in the leaves and outer leaf sheaths of resistant cultivars, indicating a potential risk of pathogen spread even in resistant cultivars. These insights have important implications for monitoring and containment protocols in *Foc* TR4-affected regions. Notably, *Foc* TR4 was not used in this study due to biosecurity restrictions.

## Data Availability

The original contributions presented in the study are included in the article/Supplementary Materials, further inquiries can be directed to the corresponding authors.

## Supplementary Materials

The following supporting information can be downloaded at: https://www.mdpi.com/article/10.3390/microorganisms12122472/s1, Figure S1: Evaluation of internal symptoms in the rhizomes of the banana cultivars tested in this study; Figure S2: Laser scanning microscopy showing the localisation of GFP-*Foc*-STR4 in the main roots of ‘GCTCV119’, ‘Williams’, and ‘Lady Finger’ at 15—45 dpi; Figure S3: Laser scanning microscopy showing the localisation of GFP-*Foc*-STR4 in the corm nodes of ‘Williams’, ‘GCTCV119’, and ‘Lady Finger’ at 26—60 dpi. (A) Mycelia in the xylem of a rhizome node in ‘GCTCV119’ at 32 dpi; Figure S4: Laser scanning microscopy showing the localisation of GFP-*Foc*-STR4 in the rhizomes of ‘Williams’, ‘GCTCV119’, and ‘Lady Finger’ at 26—51 dpi. (A) vascular coating in the rhizome of ‘GCTVC119’ at 26 dpi; Figure S5: Laser scanning microscopy showing the localisation of GFP-*Foc*-STR4 in the pseudostems and leaves of ‘Lady Finger’, ‘Williams’, and ‘GCTCV119’ at 18—65 dpi; Table S1: Reisolation of Fusarium oxysporum-like colonies from the leaves, petioles, lower sems and rhizomes of ‘FHIA02’ and ‘FHIA25’ plants inoculated with GFP-*Foc*-STR4.
