# Supplementary material for: Histological Dissection of Fusarium-Banana Interaction Using a GFP-Tagged Subtropical Race 4 Strain of Fusarium oxysporum f. sp. cubense on Banana Cultivars with Differing Levels of Resistance
Source: Microorganisms. 2024 Dec 1;12(12):2472. doi: 10.3390/microorganisms12122472 (PMC11727742; doi:10.3390/microorganisms12122472)
Supplement: Supplementary file 1 [file microorganisms-12-02472-s001.zip › microorganisms-3306178-supplementary-AC-3-Dec-2024.pdf]

Histological Dissection of Fusarium-Banana Interaction Using a GFP-Tagged Subtropical Race 4 Strain of *Fusarium oxysporum* f. sp. *cubense* on Banana Cultivars with Differing Levels of Resistance

Andrew Chen [1,*,†], Ting-Yan Chou [1,†], Yi Chen [1], Sumayyah M. A. Fallatah [1], Jay Anderson [1,‡], Jiaman Sun [1,§], Harry Cosgrove [1,‖], Siyuan Gao [1], Brett J. Ferguson [2], Amelie Soper [1], Donald M. Gardiner [3] and Elizabeth A. B. Aitken [1,*]

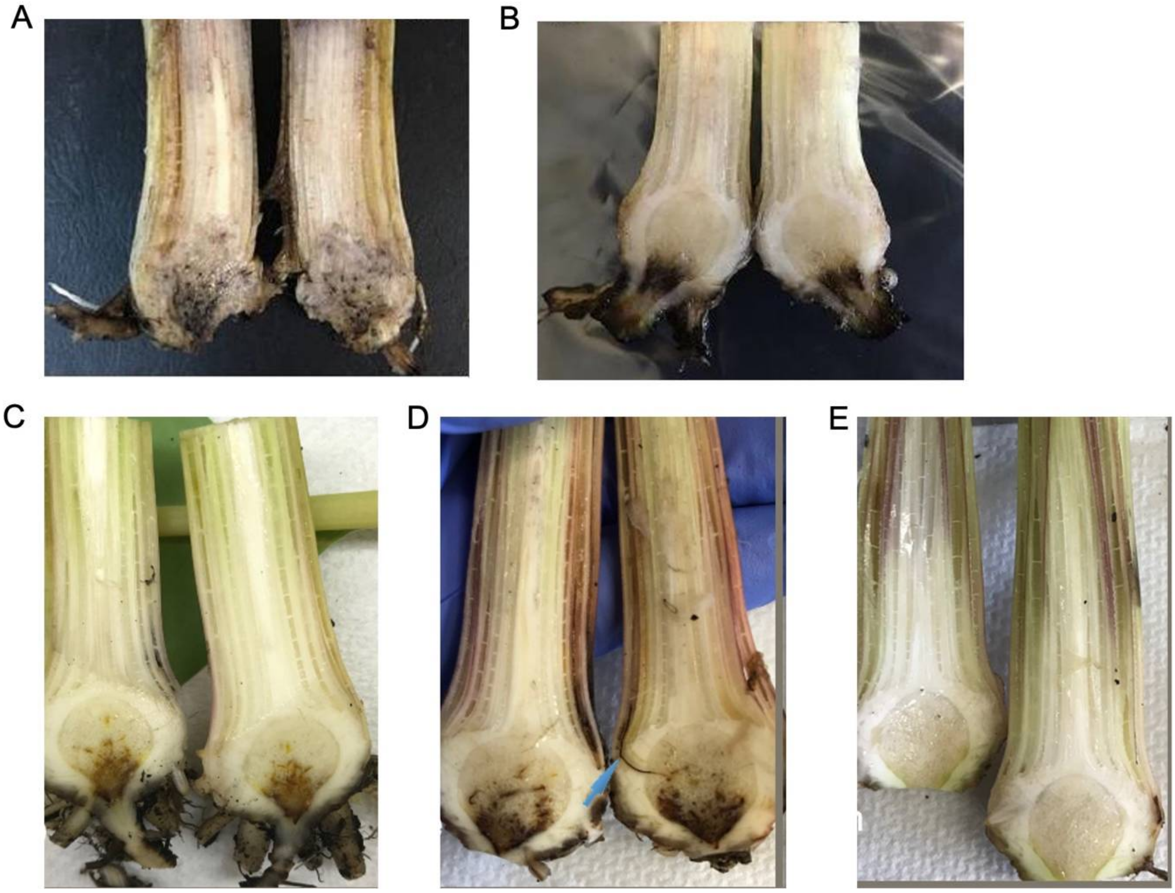

Figure S1. Evaluation of internal symptoms in the rhizomes of the banana cultivars tested in this study. (**A**) 'FHIA2' at 35 dpi. (**B**) 'FHIA25' at 35 dpi. (**C**) 'Lady finger' at 32 dpi. (**D**) 'Williams' at 34 dpi. (**E**) 'GCTCV119' at 34 dpi. The arrow points a discoloured region in the pseudostem.

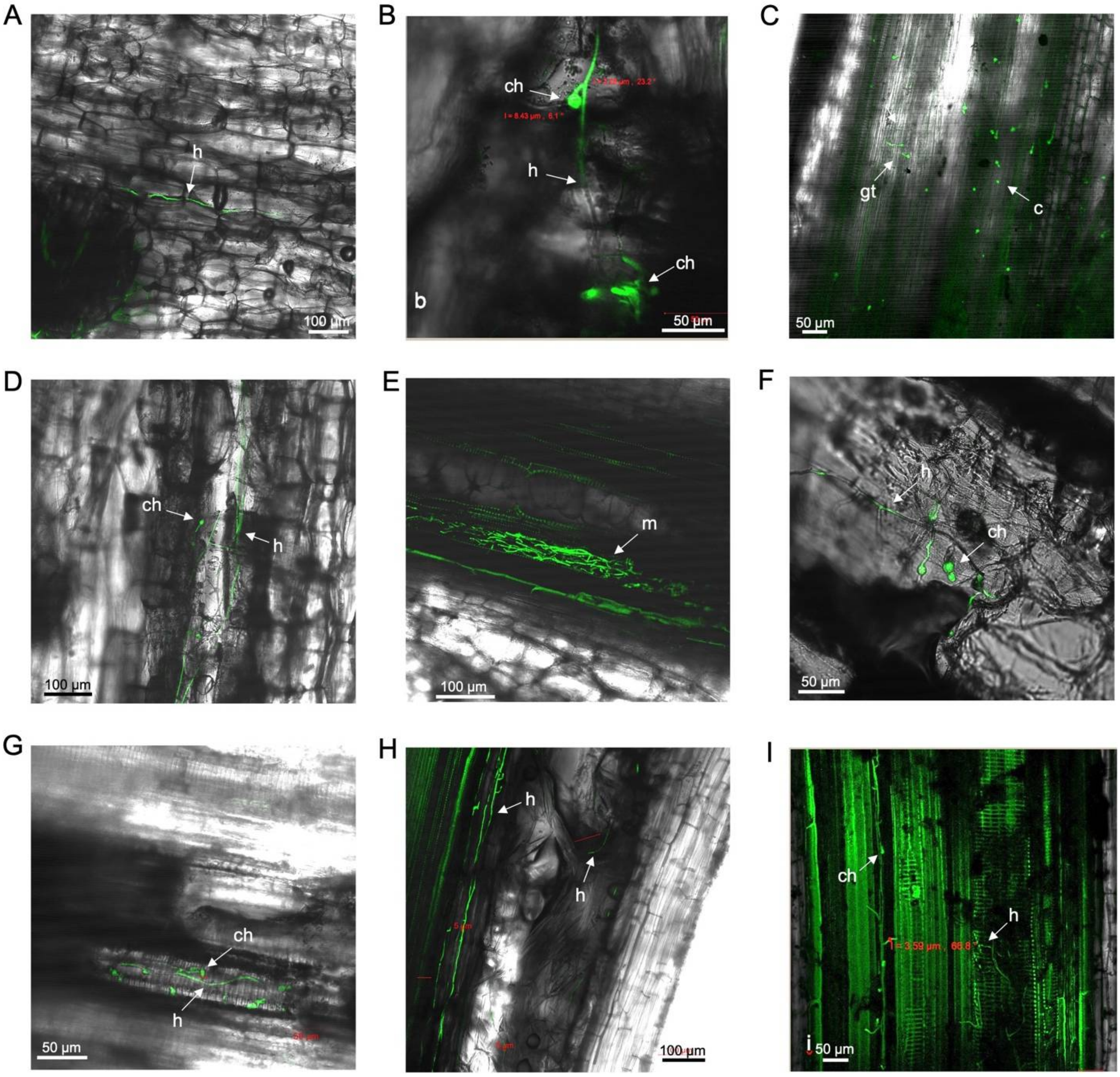

Figure S2. Laser scanning microscopy showing the localisation of GFP-*Foc*-STR4 in the main roots of 'GCTCV119', 'Williams', and 'Lady Finger' at 15—45 dpi. (**A**) Hyphae at the epidermis of the main root in 'GCTCV119' at 15 dpi. (**B**) Terminal and intercalary chlamydospores on a monophialide conidiophore in the main root of 'GCTCV119' at 15 dpi. (**C**) Germ tubes and microconidia in the main root of 'GCTCV119' at 34 dpi. (**D**) terminal chlamydospore and hyphae in the main root of 'Williams' at 27 dpi. (**E**) Mycelial networks in the xylem vessels of the main root of 'Williams' at 26 dpi. (**F**) Chlamydospores on monophialide conidiophores visualised on the main root of 'Williams' at 55 dpi. (**G**) Chlamydospores on monophialide conidophores in the xylem vessel of the main root of 'Lady Finger' at 44 dpi. (**H**) Hyphae in the xylem vesselof the main root of 'Lady Finger' at 44 dpi. (**I**) Chlamydospores and hyphae in the xylem vessel of the main root of 'Lady Finger' at 45 dpi. Abbreviations are annotated as: conidia (c); chlamydospores (ch); hyphae (h); mycelium (m); germ tube (gt). Horizontal bars indicate the scale used to capture the images.

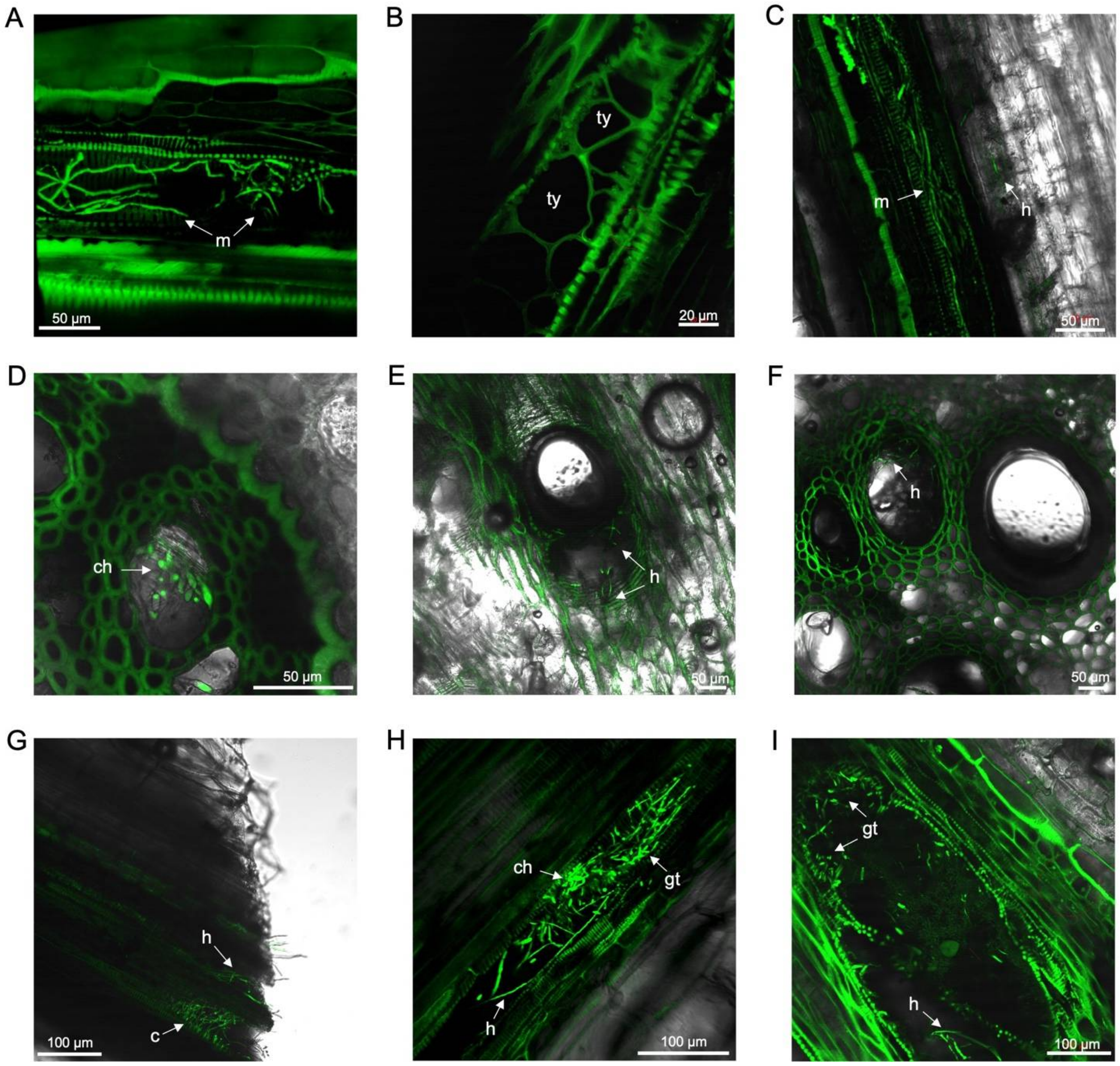

Figure S3. Laser scanning microscopy showing the localisation of GFP-*Foc*-STR4 in the corm nodes of 'Williams', 'GCTCV119', and 'Lady Finger' at 26—60 dpi. (**A**) Mycelia in the xylem of a rhizome node in 'GCTCV119' at 32 dpi. (**B**) Tyloses in a rhizome node of 'GCTCV119' at 56 dpi. (**C**) Mycelia and hyphae in the xylem vessel of a rhizome node in 'GCTCV119' at 59 dpi. (**D**) Chlamydospores in the rhizome of 'Lady Finger' at 32 dpi. (**E-F**) Hyphae in the pits of a rhizome node in 'Lady Finger' at 60 dpi. (**G**) Microconidia and hyphae in the xylem of a rhizome node in 'Williams' at 26 dpi. (**H**) Germ tubes, chlamydospores, and hyphae in the xylem vessel of a rhizome node in 'Williams' at 26 dpi. (**I**) Germ tubes and hyphae in the xylem vessel of a rhizome node in 'Williams' at 50 dpi. Abbreviations are annotated as: conidia (c); chlamydospores (ch); hyphae (h); mycelium (m); germ tube (gt); tyloses (ty). Horizontal bars indicate the scale used to capture the images.

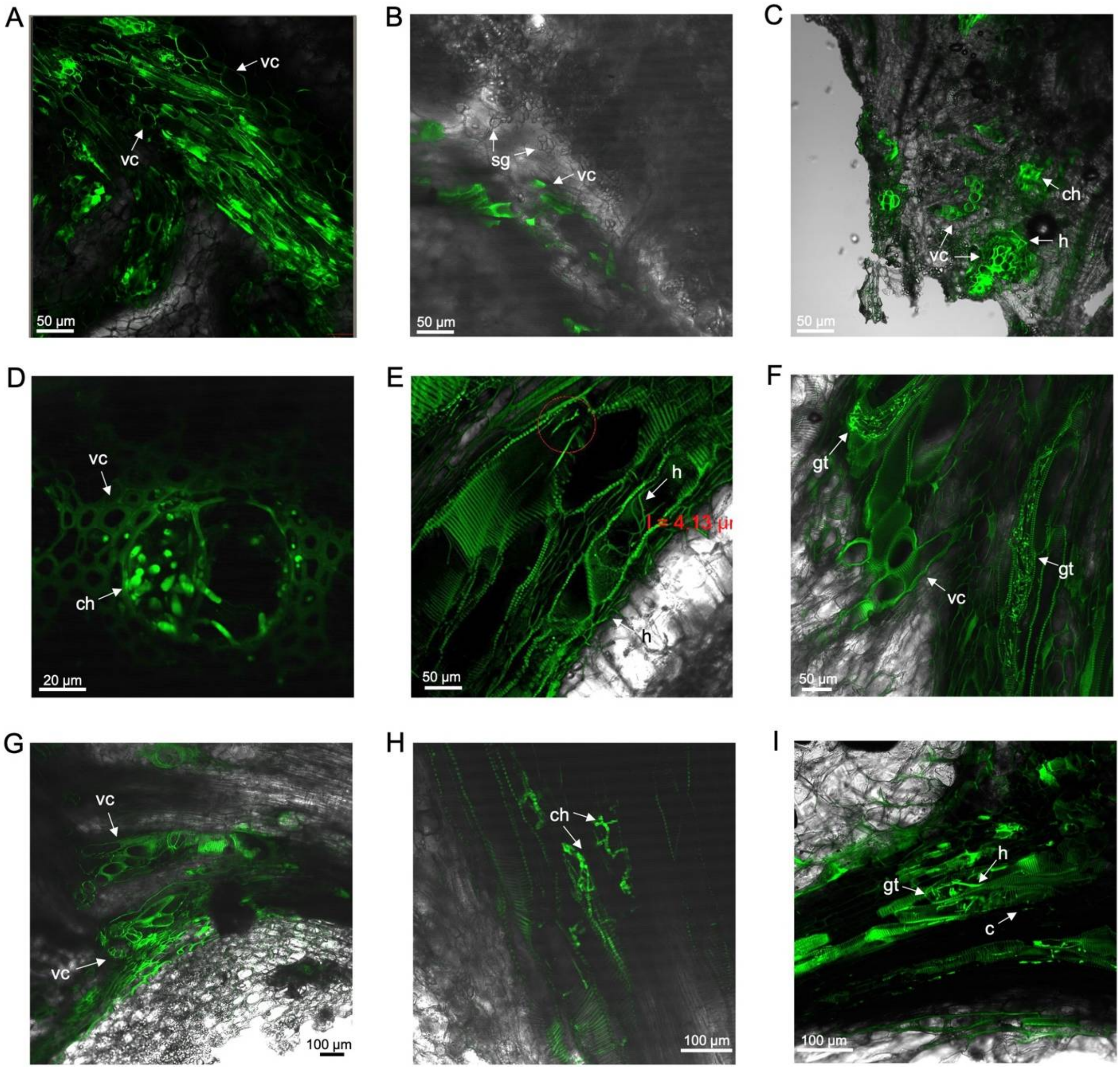

Figure S4. Laser scanning microscopy showing the localisation of GFP-*Foc*-STR4 in the rhizomes of 'Williams', 'GCTCV119', and 'Lady Finger' at 26—51 dpi. (**A**) vascular coating in the rhizome of 'GCTVC119' at 26 dpi. (**B**) Vascular coating and sugar granules in the rhizome of 'GCTCV119' at 35 dpi. (**C**) vascular coating, chlamydospores and hyphae in the rhizome of 'GCTCV119' at 42 dpi. (**D**) Chlamydospores and vascular coating in the rhizome of 'Lady Finger' at 32 dpi. (**E**) Hyphae in the xylem of a rhizome in 'Lady Finger' at 41 dpi. (**F**) Vascular coating and germ tubes in the rhizome of 'Lady Finger' at 51 dpi. (**G**) Vascular coating in the rhizome of 'Williams' at 27 dpi. (**H**) Chlamydospores in the rhizome of 'Williams' at 31 dpi. (**I**) Germ tubes, microconidia, and hyphae in the rhizome of 'Williams' at 40 dpi. Abbreviations are annotated as: conidia (c); chlamydospores (ch); hyphae (h); germ tube (gt); vascular coating (vc); sugar granule (sg). Horizontal bars indicate the scale used to capture the images.

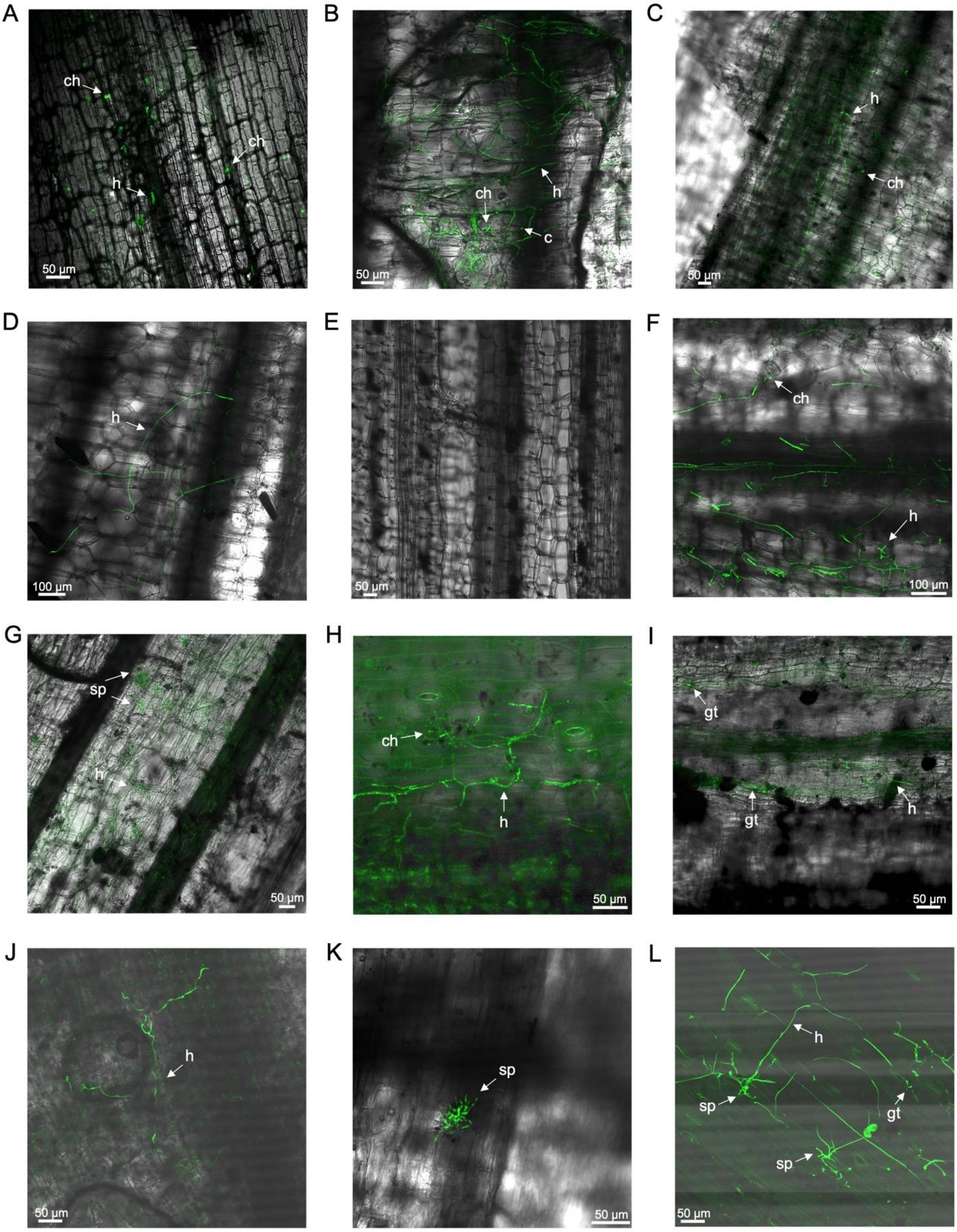

Figure S5. Laser scanning microscopy showing the localisation of GFP-*Foc*-STR4 in the pseudostems and leaves of 'Lady Finger', 'Williams', and 'GCTCV119' at 18—65 dpi. (**A**) Chlamydospores and hyphae in the pseudostem of 'Lady Finger' at 18 dpi. (**B**) Microconidia, chlamydospores, and hyphae

in the pseudostem of 'Lady Finger' at 51 dpi. (**C**) Chlamydospores and hyphae in the pseudostem of 'Williams' at 20 dpi. (**D**) Hyphae in the pseudostem of 'Williams' at 59 dpi. (**E**) Pseudostem of 'GCTCV119' in the absence of GFP-*Foc*-STR4 at 28 dpi. (**F**) Hyphae and chlamydospores in the pseudostem of 'GCTCV119' at 40 dpi. (**G**) Sporodochia on the leaf of 'Lady Finger' at 23 dpi. (**H**) Chlamydospores and hyphae on the leaf epidermis of 'Lady Finger' at 60 dpi. (**I**) Hyphae and germ tubes on the leaf of 'Williams' at 13 dpi. (**J**) Hyphae on the leaf of 'Williams' at 65 dpi. (**K**) Sporodochia on the leaf of 'GCTCV119' at 40 dpi. (**L**) Sporodochia, hyphae and germ tubes on the leaf of 'GCTCV119' at 65 dpi. Abbreviations are annotated as: conidia (c); chlamydospores (ch); hyphae (h); germ tube (gt); sporodochia (sp). Horizontal bars indicate the scale used to capture the images.

Table S1. Reisolation of *Fusarium oxysporum*-like colonies from the leaves, petioles, lower sems and rhizomes of 'FHIA02' and 'FHIA25' plants inoculated with GFP-*Foc*-STR4. '+' indicates a *F. oxysporum*-like colony with GFP fluorescence. '-' by itself indicates the absence of a *F. oxysporum*-like colony. '-' with '*' indicates the presence of a *F. oxysporum*-like colony but it was negative for GFP fluorescence when examined under a laser scanning microscope.

	GFP fluorescence																			
Plant	Leaf					Petiole					Lower stem					Rhizome				
FHIA25-1	-	-*	-*	-	-	-	-	-	-*	-*	-	-	-	-	-	-	+	+	-	-
FHIA25-2	-	-*	-	-*	-*	-*	-*	-	-	-	+	-	-	-	-	-	+	-	-	-
FHIA25-3	-*	-*	-	-	-	-	-	-*	-*	-	-*	-	-	-	-	-	-	+	-	+
FHIA02-1	-	-*	-	-	-*	-	-	-	-	-	-	-	-	+	-*	-	+	+	+	-
FHIA02-2	-*	-	-*	-*	-	-	-	-*	-	-*	+	-	-	-	-	+	+	+	+	+
